# Ranked Importance of Childhood Obesity Determinants: Parents’ Views across Ethnicities in New Zealand

**DOI:** 10.3390/nu11092145

**Published:** 2019-09-07

**Authors:** Marewa Glover, Sally F. Wong, Jacinta Fa’alili-Fidow, José G. B. Derraik, Rachael W. Taylor, Susan M. B. Morton, El Shadan Tautolo, Wayne S. Cutfield

**Affiliations:** 1 A Better Start—National Science Challenge, Auckland 1142, New Zealand; 2 School of Health Sciences, College of Health, Massey University, Auckland 0632, New Zealand; 3 TAHA Well Pacific Mother and Infant Service, University of Auckland, Auckland 1142, New Zealand; 4 Liggins Institute, University of Auckland, Auckland 1142, New Zealand; 5 Department of Women’s and Children’s Health, Uppsala University, 751 85 Uppsala, Sweden; 6 Dunedin School of Medicine, University of Otago, Dunedin 9054, New Zealand; 7 Centre for Longitudinal Research—He Ara ki Mua, School of Population Health, University of Auckland, Auckland 1743, New Zealand; 8 Centre for Pacific Health & Development Research, Auckland University of Technology, Auckland 1142, New Zealand

**Keywords:** childhood obesity, social determinants of health, cultural, ethnicity, Māori health, Pacific health

## Abstract

Māori, Pacific, Indian, and New Zealand European pre-school children’s caregivers’ views on determinants of childhood obesity are needed to inform strategies that will reduce disparities in prevalence. Nineteen focus groups were conducted to explore the relative influence of factors contributing to body weight in children. Predetermined and participant-suggested factors were ranked. Discussion data were inductively analysed. The cost of healthy foods was the highest ranked factor across all groups. Ranked similarly were ease of access to takeaways and lack of time for food preparation. Cultural factors followed by screen time induced sedentariness in children and lack of time to ensure children exercised was next. Participant-raised factors included lack of familial, social, and health promotion support, and others’ behaviour and attitudes negatively impacting what children ate. All groups rejected stereotyping that blamed culture for higher obesity rates. Compared to the Māori and NZ European groups, the Pacific Island and Indian participants spoke of losing culture, missing extended family support, and not having access to culturally appropriate nutrition education or social support and services. Public health policies need to mitigate the negative effects of economic deprivation on food insecurity. Complementary interventions that increase access to healthier meal choices more often are needed.

## 1. Introduction

Reducing the prevalence of childhood obesity is complicated by disparities among ethnicities. In the 2015/2016 year, 14.9% of 4-year-old children in New Zealand were obese, but there were marked ethnic differences in obesity prevalence [1]. Pacific children had the greatest prevalence of obesity at 30.2%, followed by Māori (20.0%), New Zealand European (12.7%), and Asian (8.1%) children [1]. This problem is not unique to New Zealand, with disparities between ethnic populations being observed in other countries. In Australia, the prevalence of obesity in kindergarten children is higher among Australian Indigenous (19%) than non-Indigenous (14%) children [2]. In the USA, Pacific Islanders, Native Hawaiians, and Alaskan Natives have disproportionately higher prevalence of childhood obesity compared to their American counterparts [3].

In addition to maternal and early life factors [4] a multitude of social and economic determinants, including ethnicity, are associated with risk factors for obesity prenatally and in early childhood [5]. For example, insufficient income strongly influences parental food choices [6]. When disposable income is limited, low-cost, energy-dense food available close to home is a pragmatic choice [7]. Although takeaway food is usually high in fat, sugar, and salt, this is an easy meal choice when there are time constraints [8]. Poor nutrition (e.g., excessive consumption of sugar, takeaway meals), children having too much screen time, lack of bedtime rules (potentially causing sleep issues), and lack of exercise have been identified as risk factors for severe obesity in children aged 5 and under [9]. Poor sleep quality and short duration of nighttime sleep are associated with some obesity-related eating behaviours [10]. Both screen time and insufficient sleep have been linked to a higher risk of childhood obesity [11,12,13]. Ethnically and culturally specific beliefs and practices may be affected by levels of acculturation, which could in turn affect childhood weight [14]. Overall, these factors overlap and can interact to impact on weight [15].

Existing interventions for preventing early childhood obesity have not been as effective as anticipated. Education has been identified as key to helping parents address childhood obesity [16]. While education is useful when nutrition literacy is low, economic and social determinants of obesity need to be considered and addressed also [7]. New information on parental views, attitudes, and beliefs about children’s weight and health is needed to inform the design of interventions that will be effective for the groups most at risk of sustained or worsening obesity with age. Research examining parental views on the determinants of childhood obesity would also improve understanding of what will have salience for parents, and how to communicate effectively with parents about weight and health in young children.

The aim of this study was to better understand how parents and caregivers of 6-month-old to 5-year-old children view the relative influence of a range of factors that might contribute to pre-school children becoming overweight. We were also interested to determine if these views differed across the main ethnic groups in New Zealand, especially in groups who have proportionately higher rates of childhood obesity.

This study is part of the A Better Start National Science Challenge (www.abetterstart.nz) programme of research aiming to increase our understanding of how parents and caregivers view growth in infants/toddlers (0–2 years) and pre-schoolers (3–5 years) and how these views influence feeding practices. This paper reports on one focus group activity that was used to facilitate discussion of factors that might influence the weight of children aged 6 months to 5 years. Thematic analysis of other focus group content which included in-depth exploration of Māori focus group findings and Pacific, Indian, and New Zealand European parents and carers is presented elsewhere [15,17].

## 2. Methods

An exploratory qualitative study design was chosen, as there had been no research carried out on parents’/caregivers’ perceptions of determinants of childhood obesity in New Zealand (NZ). Ethnic-specific focus groups were used, following strong recommendations from Pacific Island and Māori participants during the National Science Challenge stakeholder’s fora. These stakeholders suggested that focus groups would result in more in-depth explanation of parents’ views and beliefs about childhood weight. Focus groups are also useful when working with disempowered or minority groups, as the participants can provide each other with support to express culturally specific views that may be different from the perceived views of the dominant culture [18].

### 2.1. Recruitment

A non-random purposive sampling frame was used to recruit a diverse range of adult male and female parents (aged 16 and above), grandparents, or other caregivers of children aged 6 months to 5 years. Emphasis, reflected in the number of groups, was given to recruiting ethnic peoples with disproportionately high childhood obesity rates coupled with a dearth of literature on their beliefs about child weight gain. Māori, who make up 15% of the NZ population as of the 2013 Census, were a priority. Of equal importance were Pacific Island peoples, who in 2013 collectively made up 7% of the population. Pacific peoples were grouped according to Pacific ethnicity with an emphasis on running single-ethnicity groups for the more populous Pacific Island groups (i.e., Samoan, Tongan, Cook Island, and Niuean). Two pan-Pacific focus groups were also held, along with a mixed-ethnicity group of teenage mothers. Among Asian ethnic groups in NZ, Indians from India and Fiji have the highest childhood obesity rates and warranted investigation on their own. A majority of NZ citizens (74% at 2013) are of European origin. Peoples of other non-European ethnicities are often grouped with NZ Europeans, explaining the diversity in these focus groups.

Most of the participants were recruited from Auckland, the largest urban city and place of residence of the researchers, for logistical reasons. One Māori focus group was conducted in New Zealand’s northern-most city which is ranked about 9th in population size and serves a large rural region. Participants were recruited individually and through community groups. Community groups identified people from their membership or whom they served who fit the recruitment criteria and invited their participation. Some of the researchers circulated a recruitment notice among their networks of community connected workers who identified eligible individuals to make up a group. Participants were offered financial compensation for their time and travel costs in the form of a supermarket voucher.

### 2.2. Focus Group Procedure

Separate focus groups with an ethnically matched facilitator were held between December 2016 and August 2017. Using a facilitator from the same culture enabled cultural meeting protocols to be observed where required. An ethnically matched facilitator also supported participants who wanted to use their native language, although most focus groups were conducted primarily in English. Having facilitators with an understanding of the participants’ cultures mitigates “ethnocentric assumptions of meanings of other cultures” that can lead to inaccurate and incomplete representation of research results [19] (p. 1010).

Focus groups were held in rooms in school or community buildings or social or health provider rooms, convenient to the participants at a time suitable to the majority of those interested. An interview schedule was used to guide the focus group facilitation. Focus groups began with a verbal explanation of the study and distribution of a participant information sheet, consent form, and a short demographic questionnaire including the details (e.g., age, gender, relationship to child) of any children aged ≤5 years for whom care was provided. Signed consent forms and questionnaires were then collected. The entire focus group proceedings were audio recorded and subsequently transcribed. Refreshments and a light snack were provided according to cultural protocols.

This paper reports on one group activity designed to prompt discussion of the relative influence of different determinants of childhood obesity that was embedded within a wider-ranging facilitated discussion.

### 2.3. Card-Ranking Exercise

Participants were asked to rank a set of cards showing possible factors that may influence the weight of children aged 6 months to 5 years (Table 1). The initial card topics were derived from the local and international peer-reviewed literature (as at May 2016) based on discussion by the researchers, who are a mix of experts in paediatric endocrinology, childhood obesity, epidemiology, Pacific and Māori health, and nutrition. Participants were asked to rank the cards from the most to least important influence on child weight for their community. The facilitator encouraged discussion of the reasons for the ranking order. If consensus was elusive, groups were encouraged to give multiple cards the same rank order or to make a new card for important influencers they wanted to add. At the conclusion of each focus group exercise, a photo of the ranked cards was taken. The duration of each focus group was 1.5–2 h.

### 2.4. Thematic Analyses

The rank order of the cards was entered into an Excel file for analysis. Topics from new cards were entered as a new row. To contrast the relative influence of the factors on child weight by ethnicity, the rank given to each factor was averaged across each ethnic group—Māori, Pacific, NZ European (including the teenage parents group), and Indian. The factors were colour-coded and tabled to illustrate the average rank of the factors for each of the four ethnic groups.

The complete list of factors, both those provided at the start and new cards created by the groups, was then categorised by two of the researchers (M.G., S.F.W.) in order to group cards with related content. For example, several created cards were about not having enough time to prepare healthy foods, and these were grouped together as a new theme. When a new card’s meaning was not clear, the relevant transcript was searched for discussion that would explain the intended meaning of the factor. The remainder of the transcribed discussion about the exercise that did not fit within the categories provided by the cards was inductively grouped to form additional themes [20], which were then used to support and validate the results of the ranking exercise. Illustrative quotes were drawn from the transcripts.

### 2.5. Ethics Approval

This study was approved by the University of Auckland Human Participants Ethics Committee (#018082). All participants provided both verbal and written informed consent. Questionnaires and focus group transcripts were anonymised prior to analysis.

## 3. Results

Nineteen focus groups with a total of 180 participants were conducted. The ethnic composition of the groups is summarised in Table 2 and overall demographic data are presented in Table 3. Most participants were females (78.3%); 43.9% identified as one of several Pacific ethnicities, equal percentages as Māori (22.2%) or NZ European (22.2%), and just under half were born overseas (46.7%). Approximately half of participants were aged 30 to 50 years (52.9%) and just over a quarter were under the age of 30 (26.7%).

The 10 original factors influencing child weight (as shown in Table 1), in order of their relative perceived influence for each ethnic group, are shown in Table 4 (with “Culture” being a composite of “big is seen as beautiful”, “culture encourages eating”, and “events and festivals”). This ranking highlighted that the cost of food was unanimously chosen by all groups as the most important factor, with the easy access of takeaways (Māori, Pacific), too much screen time (Pacific, Indian), and culture (NZ European/Other) being ranked second by different groups. New cards created by the participants expanded the original list from 10 factors to 56, which were reduced to the 13 themes shown in Table 5 by thematic analysis. Each of these 13 themes are discussed in the following sub-sections in order of the relative ranking or similarity of theme.

### 3.1. Cost

The cost of healthy foods was perceived to be the strongest influence on children’s weight by all ethnic groups. This factor was closely related to focus group discussions of cheaper foods, which were considered to be “unhealthy” (e.g., takeaways, fatty cuts of meat).

### 3.2. Convenience

“Takeaways are easier to access” was perceived to be a different factor from cost because convenience is more related to time. Māori and Pacific groups identified the card “takeaways are easy to access” to be the second most important influence on child weight, with Indian and NZ European groups ranking it third. Related to this was that participants reported that they lacked access to quick, cheap, and healthier options.

### 3.3. Time

Indian parents and caregivers ranked lack of time as a first equal factor influencing child weight, along with cost. Māori and Pacific groups identified it as third most important and NZ Europeans ranked it fifth. Along with the provided card “time to prepare healthy foods”, several groups created new cards expanding on this theme. These included cards for “working/busy parents”, “not enough time to spend with kids” (e.g., cooking, feeding, quality time), and “long waiting time at doctors”.

### 3.4. Taste

Multiple groups created taste-related cards, reflecting the perceived influence of their children’s food preferences. Several groups created a card labelled “fussy eaters”. Other new cards included “taste of healthy foods” (i.e., kids did not like the taste of healthy foods) and “taste, i.e., sweet, salty” (i.e., kids have preferences for sweet or salty foods, for example).

### 3.5. Eating Too Much

The cards “portion sizes” and “energy in > energy used” were grouped together, as in both cases, parents considered eating too much to be an important factor contributing to excess weight.

### 3.6. Physical Inactivity

Participants did not add any cards about physical inactivity, but four cards identified in the literature review were included in this theme. Both Pacific and Indian parents/caregivers considered “too much screen time” to be very important, ranking it as the second most important factor influencing child weight (Table 4). Compared to this, NZ Europeans and Māori ranked screen time fifth and seventh equal (last), respectively.

“Lack of exercise” (referring to the card “Not enough time to exercise”) was ranked fifth by Māori, Pacific, and Indian groups. NZ European considered this factor to be sixth equal as a contributor to children’s obesity. Similarly, “poor sleep” was listed as a contributing factor by all four groups: Indian (third equal), Pacific (fifth equal), Māori (sixth), and NZ European (sixth equal). While many groups talked about sports or exercise activities for kids being too expensive, the “cost of sport” card was ranked the lowest relative to other key factors.

### 3.7. Cultural Factors

Three factors related to culture were derived from the literature review—“big is seen as beautiful”, “culture encourages eating”, and “events and festivals”. No additional cultural factors were identified by participants.

Culture was ranked highly by all groups, with NZ European parents/caregivers ranking it second and Māori, Pacific, and Indian groups ranking it fourth. However, while culture was acknowledged as an important influence on determining a child’s weight, many participants made it clear that culture was not a bad thing. For example, a card for “traditional versus Western food” suggested that it was the Western influences that were inhibiting a parent’s ability to provide a healthier diet to their children. Some healthy-eating alternatives were presented in opposition to the “culture encourages eating” card. Notably, this card was deemed offensive to some participants because it was seen as inappropriate in its deficit framing of culture. Across the Pacific, Māori, and Indian groups, participants said that actually all cultures have values around food that are positive. One example given in a Māori focus group was *manaakitanga* (showing respect and care by being a good host and looking after guests, including providing them with drink and food). Similarly, Pacific participants talked about *fatongia* (the obligation to provide and the obligation to eat what is provided). Both of these were seen as positive cultural values related to food.

### 3.8. Spirituality

Spirituality (Table 5) was unable to be grouped with any other cards. It was identified as an important influencing factor by a Tongan group. As described by one participant, “if we throw in spirituality, we have a responsibility to look after them if their body is a temple.”

### 3.9. Social Influence

Social influence (Table 5) was a new theme derived from a subgroup of cards created across a number of focus groups. Māori groups wanted it recognised that while they might be trying to feed their children healthy food, other influencers had “opposing views”. The Pan-Pacific, Niuean, and NZ European groups also created a new card for the same barrier, describing that other family members, such as grandparents, gave unhealthy food to children. The Tongan group labelled it “family structure”, which encompassed extended or multiple families living together, referring to other people who participated in feeding children. This group discussed that unless “everyone” was “on the same page”, children might be given, for example, “fizzy drink”.

### 3.10. Parents’ Problems

Some groups created cards that framed problems that parents have as influential factors, such as “lazy parents”, “uneducated parents”, “emotional wellbeing of parents”, and “parents with other priorities, e.g., addictions, partying”. The Tongan group and the NZ European fathers group both identified lack of routine as having a negative influence on the weight of children.

### 3.11. Lack of Support

Lack of support was related to a lack of time, but there were several cards that were more specifically about lack of support per se. A Māori group, an Indian group, and the Tongan groups independently created a card for “lack of support”. Explanations given included that the people around the parents might not be supportive, or that no support was available due to isolation. For example, an Indian group talked about being deprived of parental and other family support due to migrating to NZ as a single family, as opposed to an extended family unit:

- “Here [in NZ] the atmosphere is very different—single family, single child—not like in India where we have family, cousins, granny, and big support there.”

The Cook Island group said that eating healthy foods requires support from the whole family, that it should not be just “the children doing it, but kind of doing it more together being supportive of each other.” Similarly, they said that providing healthy food needed the whole family to assist in the food preparation because of the time involved, as summarised by one participant:

- “Preparation is hard. ‘Cause you know what, sometimes you’re doing all the cooking and you’re pretty much too tired to eat after, ‘cause you’ve done so much prep to prepare all this food. It’s just going to be gone in a matter of minutes.”

All groups stressed that there were many demands and pressures on parents and families which undermined their ability to feed children healthy foods and ensure the children got sufficient physical exercise. Lack of support for dealing with these other pressures meant that the time, effort, stress, and energy required to plan, prepare, and serve healthy foods was too much for them to cope with. One of these stressors was “housing and environment”:

- “We’ve got housing conditions and environment… in terms of hygiene and if there’s overcrowding and dampness, that contributes to their weight because if they’re sick all the time they’ll lose weight.”

One source of support that could have helped alleviate other pressures (let alone provide more time) was childcare, with a Māori group creating the card “childcare is expensive”.

### 3.12. Loss of Culture

Loss of culture was a new theme that was derived from a subgroup of cards created across several focus groups. Cards amalgamated under this theme were, in essence, produced by participants who commented on the loss of aspects of their culture. Cards included “lack of access to culturally appropriate information and resources” and “culturally appropriate services” (Indian groups) and “extensive cooking time needed for Indian cooking”, “traditional versus Western foods”, “economic manipulation of Pasifika culture/group psychology”, and “loss of skill and knowledge to grow own food/knowing how to garden”.

The Pacific fathers group said that their card “economic manipulation of Pasifika culture/group psychology” was about fast food retailers deliberately exploiting positive Pacific values by positioning cheap food outlets in their communities:

- “like those debt trucks, and even things like liquor stores that you don’t see over at St. Heliers [a high socioeconomic suburb], things like all of the fast food, really fatty food takeaways that you don’t see over in some of the other areas of Auckland, they’re all just here… When you go to [low socioeconomic suburb], the first thing you look at is, you know, all the takeaways are open and Subway’s closed, we don’t even have a choice not to go there… You don’t have the quality shops here for the community itself.”

As mentioned in a previous section, “traditional versus Western foods” was grouped with other cards that suggested that it was Western influences that were inhibiting the provision of a healthier diet to children. As explanation, a participant said that

- “In Samoa, all the generations eat the same food (no separate meals for children and adults). But when we come to NZ, it’s too much food, too much takeaway, too much drink.”

### 3.13. Nutrition Literacy

Varying levels of literacy about food and nutrition, along with conflicting information, were identified as barriers to optimal food provisioning. Parents and caregivers mentioned having a limited range of cooking skills, having limited recipe knowledge, and not knowing how to cook different foods, especially different types of salads:

- “So, we can make coleslaw and green tossed salad, but all the other things like couscous and all that kind of stuff—quinoa, we don’t know those kind of stuff so it looks expensive and we’re not exposed. We just know how to put the basics: spinach and cabbage into the corned silverside. That’s the vegetables. That’s how we do vegetables. We don’t really do like, other types of salads that’s out there.”

The NZ European group felt there was a lot of pressure on parents to provide the “right” foods, exercise, and lunch boxes, which in turn created more stress for parents. A participant in the Indian group also spoke about the stress caused by conflicting information:

- “Confusion about information available due to generation gap, cultural gap, system gap, lifestyle, and different beliefs... Conflicting views which can become difficult. Confusion about information available due [to] white culture, from elders back home, and due to generation gaps. System gap—system does not understand our beliefs, practices, boundaries, and other influences in our life and upbringing. Main system assume that we know all as they do.”

Participants in different groups mentioned a range of ideologies around food, such as “clean eating”, “sustainability”, and “spray free… free range, grass fed”.

## 4. Discussion

This was a large multi-ethnic qualitative study involving 180, mainly female, parents and caregivers. Across all the participating ethnic focus groups, the most influential factor perceived to impact on child weight gain was the relatively higher cost of healthy foods, followed by the ease of access to takeaway meals. Extensive discussion through ethnic-specific focus groups yielded many additional relevant factors, not initially part of the ranking exercise, highlighting the richness of data obtained through such techniques. Eating behaviours such as taste preferences and portion sizes were considered major determinants of child weight, as were social influences including peer pressure, the influence of other family members on the diet of children, and how the parents themselves ate. Parents identified several non-food-related issues that placed additional demands and stresses on families that in turn undermined their ability to provide a healthy lifestyle for their children, such as the housing environment, the expense of childcare, and inability to have a good work/life balance. Finally, issues with health or food literacy and the array of conflicting information about food and health from difference sources made it difficult to know what other options there were for feeding children healthy foods.

The association of diet disparities with socioeconomic status is well established [21,22]. Childhood obesity rates have also been associated with socioeconomic status and residence area level [23], or home-based [24], deprivation. In New Zealand, the prevalence of obesity among children residing in areas of greatest socioeconomic deprivation (22.4%) is more than twice that of children from the least deprived areas (9.7%) [1]. Further analysis by Shackleton et al. (2019) [23] suggests that reducing socioeconomic inequalities would have a large impact on reducing the difference in obesity rates between Māori and European/Other and between Pacific and European/Other children. There is some controversy as to whether healthy foods are more expensive than takeaway meals in New Zealand (e.g., [25]), but such critiques fail to take into account broader pressures such as time scarcity and structural societal inequities that create food insecurity and differential access to recommended foods [7,26]. Our deeper analysis of the complete data set from the Māori focus groups suggested a complex interdependence between multiple social and economic determinants of health, requiring a systems or holistic understanding [15].

The three lowest ranked influencing factors overall—lack of exercise, poor sleep, and cost of sport—might have been relegated to the bottom of the ranking due to poor wording used on the cards. Some participants questioned how relevant these were given the age range being focused on. For example, children aged five years and under do not typically participate in sport or exercise. Thus, in hindsight, these two cards could have been reframed as insufficient physical activity. Poor sleep was not perceived to be a problem, except among Indian families. Similar to our findings, screen time was also not identified as an important determinant of excess weight in children among Alaskan Natives in the Children’s Healthy Living project [3].

The cards that presented statements such as “culture encourages eating”, which was established early on in New Zealand obesity literature and became a popular belief [27,28], were rejected by many participants as offensive and deficit focused. Some Pacific groups, but not Māori groups, also disagreed with the card “big is seen as beautiful” which represented the stereotyped belief that “a fat baby is a healthy baby” [29]. For some Pacific and Indian groups, loss of culture due to integration into New Zealand society or a shift from traditional foods and the practices and meanings associated with traditional foods, such as all members of the family eating the same dish, was identified as a negative influence on weight gain. This is beginning to be explored in other studies, such as Tupai-Firestone et al. [30], whose study with Pacific youth found that a large proportion retained strong cultural identify whilst being adapted to Western lifestyle. They concluded that it is not necessarily a positive trajectory if this results in the loss of culture as a health resource [30]. Further research is needed into both Pacific Island and Indian experiences of change in their diets due to migration.

This study only investigated the relative importance of perceived determinants of childhood obesity. Other parental concerns about their children’s growth and health were not considered. In their study of Australian kindergarten children, Hickie et al. (2013) found that only 12% of parents were concerned about their child becoming obese [2]. In the United Kingdom, only 6% of parents viewed their children as overweight and none of them described their child as “very overweight”, despite 145 out of 564 children being assessed as overweight or obese [31]. There was no association between this perception and demographic variables such as parental education, age, sex, or ethnicity [31]. However, that cohort was 72% Caucasian, which limits the ability to assess potential ethnic differences.

### 4.1. Strengths and Limitations

One strength of the study was the use of a parallel method involving ethnic-specific focus groups. Having ethnically matched facilitators who encouraged culturally specific views to be expressed likely mitigated a number of biases, such as social desirability bias and culture bias that favour dominant-culture perspectives [32], resulting in many possible influences on child weight being suggested by the participants themselves. However, the use of a range of facilitators introduced variance in how the focus group procedures were conducted, which was a limitation (e.g., some facilitators seem to have prompted deeper explanations than others).

Another strength of this study was the involvement of grandparents and other caregivers in our focus groups, which added to the diversity of views presented.

One limitation was that fathers and other male caregivers were under-represented. In addition, the majority of our participants lived in Auckland, New Zealand’s largest city. While one focus group was conducted in Northland, most participants lived in urban areas. Thus, the results may not be generalisable to families living in other regions, and particularly to those living in small towns and rural areas.

Moreover, because this study relied on ranking, there is a risk that this method might have exaggerated the difference between the perceived influences of the various factors. Nonetheless, a strength of the ranking approach is that factors are assessed relative to each other. The advantage is that more of the context is considered when ranking, as opposed to rating individual factors on a scale and in isolation. This is relevant because a related study showed that parents and caregivers weigh multiple interacting factors when considering what food to provide to their children throughout each day [15]. The authors recommended consideration of the interdependent determinants when designing interventions rather than focusing on single influencing factors [15].

### 4.2. Recommendations for Future Research and Policy

Unaffordable healthy food, time scarcity, and convenient access to fast and affordable meal choices close to home present challenges for designing interventions to reduce childhood obesity. While parents/caregivers of young children want to fulfil their parenting role and intend to feed their children well, the competing demands on their time, tiredness from work, and lack of support for food preparation or childcare means that they often felt that they had little choice but to buy convenient readymade meals from takeaway food shops close to home.

Notably, the ethnic groups in our study described different needs. For example, Indian participants reported a lack of culturally appropriate information, while the Pacific groups reported a need for education on the wider range of foods available in New Zealand (e.g., quinoa) and how to cook them.

As recommended by Glover et al. [15], Fialkowski et al. [3], and Graham et al. [7], more holistic interventions that consider the wider social and environmental determinants of childhood obesity need to be developed with input from the affected communities. Increasing parents’ nutrition literacy and efficacy with a range of cooking methods is one suggestion. Another observation from the focus groups is that the purchase of less healthy fast-food meals could be reduced if there were conveniently located healthier meal choices available for parents. The evidence provided by our participants suggests that these options simply do not exist in socioeconomically deprived areas.

It is important to identify solutions that do not unintentionally worsen the barriers to healthier eating by increasing the time involved and/or the cost of preparing meals or stigmatisation and guilt resulting from feeling like the food being provided is judged to be “wrong”. Interventions that focus on education risk stereotyping parents as deficient, and these interventions will have little salience to parents who are simply unable to provide healthier food due to financial constraints [7]. Public health policies that aim to reduce the economic and social determinants of food insecurity and that increase parents’ access to affordable healthier foods are likely to have higher attractiveness for the populations represented in this study: Māori, Pacific, and lower socioeconomic parents. Complementary educational and supportive strategies to increase parents’ access to heathier meals more often are needed.

## Figures and Tables

**Table 1:** List of provided pre-printed cards showing possible reasons affecting weight of infants and young children.

• Big is seen as beautiful
• Cost of healthy foods
• Culture encourages eating
• Events and festivals
• Has trouble sleeping
• Not enough time to exercise
• Sports are too expensive
• Takeaways are easy to access
• Time to prepare healthy foods
• Too much screen time

**Table 2:** Ethnic composition of focus groups, showing the respective numbers of participants.

Focus Groups	Actual Ethnic Composition of Individuals
Māori (5 groups)	32 Māori, 2 Samoan, 2 Tongan, 1 Cook Island Māori
Pacific (6 groups)	64 Pacific
NZ European (4 groups)	34 NZ/other European, 1 Chinese, 1 Vietnamese
Indian (3 groups)	28 Indian, 5 Indo-Fijian, 2 NZ European, 1 Pakistani
Teenage parents (1 group)	4 NZ/other European, 2 Māori, 1 Samoan

**Table 3:** Demographic profile of study participants (*n* = 180).

Demographic Characteristic	Groups	
Sex	Female	141 (78.3%)
	Male	39 (21.7%)
Age group (years)	<20	6 (3.4%)
	20–29.9	41 (23.3%)
	30–39.9	64 (36.4%)
	40–40.9	29 (16.5%)
	≥50	36 (20.5%)
Ethnicity *	Māori	40 (22.2%)
	Samoan	32 (17.8%)
	Tongan	22 (12.2%)
	Cook Island Māori	14 (7.8%)
	Niuean	11 (6.1%)
	Indian	27 (15.0%)
	NZ European	40 (22.2%)
	Other European	5 (2.8%)
	Other	14 (7.8%)
Country of birth	Not in New Zealand	84 (46.7%)
	If not, years lived in NZ (median (IQR))	11 (1–54)
Number of other persons in household	Adults (mean (range))	3 (0–9)
	Children (mean (range))	2.5 (0–10)
Education	No school qualification	23 (12.9%)
	High-school qualification	46 (25.8%)
	Post-school qualification (trade, diploma or certificate)	55 (30.9%)
	University Degree	54 (30.3%)
Marital status	Single/never married	32 (18.0%)
	Married/de facto/civil union	123 (69.1%)
	Widowed	6 (3.4%)
	Separated/divorced	17 (9.6%)
Employment status	Student	22 (12.4%)
	Homemaker	50 (28.1%)
	Full-time or part-time employed	72 (40.4%)
	Retired	14 (7.9%)
	Not currently employed	20 (11.2%)
Partner’s employment status	I do not have a partner	34 (19.3%)
	Student	2 (1.1%)
	Homemaker	12 (6.8%)
	Full-time or part-time employed	102 (58.0%)
	Retired	9 (5.1%)
	Not currently employed	17 (9.7%)
Relationship to child	Parent	123 (68.3%)
	Grandparent	24 (13.3%)
	Step-parent	1 (0.6%)
	Aunt or uncle	10 (5.6%)
	Other	4 (2.2%)
	Not disclosed	18 (10%)

Unless otherwise stated, data are *n* (%). * Participants could nominate more than one ethnicity. IQR, interquartile range.

**Table 4:** Relative influence of factors on child weight across the ethnic groups.

	Māori		Pacific		Indian		NZ European/Other	
1	Cost of Healthy Food		Cost of Healthy Food		Cost of Healthy Food	Time	Cost of Healthy Food	
2	Takeaways		Screen Time	Takeaways	Screen Time		Culture	
3	Time		Time		Takeaways	Poor Sleep	Takeaways	
4	Culture		Culture		Culture		Time	
5	Lack Exercise		Lack Exercise	Poor Sleep	Lack Exercise		Screen Time	
6	Poor Sleep		Cost of Sport		Cost of Sport		Poor Sleep	Lack Exercise
7	Screen Time	Cost of Sport					Cost of Sport	

**Table 5:** Factors influencing child weight according to New Zealand parents and caregivers by theme.

Theme	Factors
Cost	Cost of healthy foods Takeaways are cheaper Cheaper food is often unhealthy, e.g., fatty cuts of meat
Convenience (takeaways)	Takeaways are easier to access
Time poor	Time to prepare healthy foods Working/busy parents Not enough time to spend with kids, e.g., cooking, feeding, quality time Waiting time at doctors
Taste	Taste, i.e., sweet, salty Taste of healthy foods Fussy eaters
Eating too much	Portion sizes Energy in > energy used
Physical inactivity	Too much screen time Has trouble sleeping/lack of sleep Not enough time to exercise Sports are too expensive
Cultural factors	Culture encourages eating Events and festivals Big is seen as beautiful
Spirituality	
Social influence	Others’ opposing views What other children eat, i.e., peer pressure Family structure/Other family members giving food, e.g., grandparents How parents eat/What you enjoy influences your children
Parents’ problems	Lack of routine Lazy parents Uneducated parents Parents with other priorities, e.g., addictions, partying Emotional wellbeing of parents
Lack of support	Food preparation involving whole family’s assistance Children participating Childcare is expensive Supporting healthy eating as a family Housing and environment Lack of balance: work, life, and care for my child Lack of access to culturally appropriate services Pressure/stress on parents to provide the “right” foods and exercise
Loss of culture	Lack of access to culturally appropriate info and resources Extensive cooking time needed for Indian cooking Traditional vs. Western foods Economic manipulation of Pasifika culture/group psychology Loss of skill and knowledge to grow own food/Knowing how to garden
Nutrition literacy and conflicting information	Lack of health literacy Lack of food literacy Healthy, affordable food options, e.g., different types of salads, cooking methods Cooking skills Parental guilt Mindset—ideology around food Confusion about information available due to generation gap, cultural gap, system gap, lifestyle, and different beliefs.
